# A decade of child pedestrian safety in England: a bayesian spatio-temporal analysis

**DOI:** 10.1186/s12889-023-15110-2

**Published:** 2023-02-01

**Authors:** Niloofar Shoari, Shahram Heydari, Marta Blangiardo

**Affiliations:** 1 MRC Centre for Environment & Health, Department of Epidemiology and Biostatistics, Imperial College London, London, UK; 2 Transportation Research Group, Department of Civil, Maritime, and Environmental Engineering, University of Southampton, Southampton, UK

**Keywords:** Bayesian spatio-temporal, Child pedestrian safety, Road crashes, Hierarchical models

## Abstract

**Background:**

Child pedestrian injury is a public health and health equality challenge worldwide, including in high-income countries. However, child pedestrian safety is less-understood, especially over long time spans. The intent of this study is to understand factors affecting child pedestrian safety in England over the period 2011–2020.

**Methods:**

We conducted an area-level study using a Bayesian space-time interaction model to understand the association between the number of road crashes involving child pedestrians in English Local Authorities and a host of socio-economic, transport-related and built-environment variables. We investigated spatio-temporal trends in child pedestrian safety in England over the study period and identified high-crash local authorities.

**Results:**

We found that child pedestrian crash frequencies increase as child population, unemployment-related claimants, road density, and the number of schools increase. Nevertheless, as the number of licensed vehicles per capita and zonal-level walking/cycling increase, child pedestrian safety increases. Generally, child pedestrian safety has improved in England since 2011. However, the socio-economic inequality gap in child pedestrian safety has not narrowed down. In addition, we found that after adjusting for the effect of covariates, the rate of decline in crashes varies between local authorities. The presence of localised risk factors/mitigation measures contributes to variation in the spatio-temporal patterns of child pedestrian safety.

**Conclusions:**

Overall, southern England has experienced more improvement in child pedestrian safety over the last decade than the northern regions. Our study revealed socio-economic inequality in child pedestrian safety in England. To better inform safety and public health policy, our findings support the importance of a targeted system approach, considering the identification of high-crash areas while keeping track of how child pedestrian safety evolves over time.

**Supplementary Information:**

The online version contains supplementary material available at 10.1186/s12889-023-15110-2.

## Background

Road safety is a global public health concern and one of the leading causes of death for children over the age of 5 years [1, 2]. Child pedestrians are particularly vulnerable road users due to their limited physical, cognitive-perceptual, and social development [3]. According to the UK Department for Transport, only in 2019 in England, 4700 child pedestrians under the age of 15 sustained traffic-related injuries, out of which 1200 were killed or seriously injured. Ensuring children’s safety on roads is a major public health priority as it can prevent various adverse physical, mental, and social consequences and can promote walking among children, increasing childhood physical activity.

Adopting active modes of travel, including walking and cycling, from early ages has positive impacts on both personal and planetary health. However, the number of children walking has in general declined over the last decades. For example, based on the National Travel Survey statistics, in England in 1998/2000, an estimated 49% of children under 16 walked to school while in 2017 this rate fell to 43%. Research suggests that this reduction is partly due to traffic safety concerns [4, 5]. To help address this issue and reverse this trend, it is essential that policies are geared towards making roads safer and enjoyable for children.

Child Pedestrian injury is a multi-faceted problem with many contributory factors such as driver characteristics [6], vehicle features [7, 8], road configuration [9, 10], environmental [11], built-environment [12], and socio-economic and ethnic features [13–18]. In addition to these factors, there are geographical and temporal variations influenced by place- and time-specific factors and interventions such as reduced vehicles circulations around schools, implementation of 20 mph speed limit zones, school walking bus, School Street schemes, among the others.

Previous research has mainly focused on identifying high-crash areas and linking the risk to explanatory variables to quantify the effect of several risk factors through a combination of geographic information systems and statistical models [19–24]. Due to the nature of road crash data, there might be spatial and temporal dependencies between observations; and therefore, statistical models need to accommodate these dependencies [25]. However, considering spatial-temporal dependencies [26] as well as child safety [15] is relatively limited in the crash literature, especially in England. In this study we investigate the spatiotemporal patterns of child pedestrian safety at Lower Tier Local Authorities (LTLA) level in England from 2011 to 2020. In England, the LTLA is a subdivision of administrative boundaries that include local authority districts, unitary authorities, metropolitan districts, and London boroughs. The main aims are to (i) explain the association between child pedestrian crash frequencies and LTLA level characteristics, including a host of deprivation, transport, and built-environment variables, (ii) identify LTLAs with particularly high crash frequencies for child pedestrians across the study period, (iii) evaluate the persistence of spatial patterns of child crashes over time, and (iv) pinpoint local time trends for each LTLA.

We conducted our analysis within a Bayesian framework as the Bayesian approach is particularly appealing in handling spatio-temporal dependencies in the data, readily allowing for borrowing of strength across space and time that leads to more reliable estimates. The Bayesian approach, unlike the frequentist approach that obtains point estimates of parameters of interest, regards the unknown parameters as random variables and obtains posterior densities for all model parameters, addressing uncertainties more fully. For a discussion on the advantages of the Bayesian methods in the context of road safety analysis, see [27]. The posterior density is the result of data (i.e., the likelihood function) integrated with a prior probability distribution that represents our prior belief regarding a parameter of interest. When there is no prior knowledge of a parameter, non-informative priors can be used, allowing the data to decide the form of the posterior entirely. For estimating the model parameters, the Bayesian approach employs Markov chain Monte Carlo simulations [28].

## Methods

### Data

The outcome of interest was the annual counts of crashes involving child pedestrians at local authority level in England. Crash data were obtained from the Department for Transport, which collects information on crashes that occurred on public roads, reported to the police, and recorded on STATS19 forms. As the focus of our study was to model child pedestrian crash frequencies, information relating to the location of crashes, road user types (e.g., pedestrian, cyclist, driver), and age of individuals involved in crashes were extracted from the STATS19 databases. We only included crashes between a motorised vehicle and a child pedestrian, who was less than 16 years old. This yielded 50,993 crashes over the period 2011–2020. Using the geographic coordinates of the crashes, we obtained yearly crash counts at LTLA level. We removed Isles of Scilly due to the sparsity of outcome data and the City of London because many explanatory variables for the latter were missing. Isle of Scilly and the City of London had one and ten cases of child pedestrian crashes over the study period, respectively. This resulted in 315 LTLAs with a mean crash of 161, standard deviation of 169.83, minimum of 6, and a maximum of 1808 over the study period. The geocoded location of crashes is mapped in Additional File 1.

To model child pedestrian crash frequencies, we considered several sociodemographic, transport, and built-environment features, based on literature, domain expertise, and data availability. As sociodemographic covariates, we considered percent of child (0–15 year) population [29], number of licensed vehicles per capita [30], percent of claimants, percent population who are White, and job density [31]. The number of licensed vehicles in 2020 per capita was calculated by dividing the total number of registered vehicles by local authority population. The percent of claimants for 2011–2020 was defined as the proportion of residents aged 16–64 claiming some form of unemployment-related benefit. From the annual population survey [31], we obtained yearly unemployment rate, and yearly percent of population in employment who are manager, directors, and senior officials for 2011–2020.

As transport-related variables indicating travel behaviour and exposure at LTLA level, we included the percent of adults who walk or cycle at least three times per week, the percent of adults who walk or cycle at least five times per week [32], and road density. To obtain road density, the latest road network data sourced from Ordnance Survey Meridian [33] was overlaid with LTLA boundaries. We then calculated road density by dividing the total length (in km) of A roads and B roads by the total land area (in km^2^) of each LTLA. The A roads are major roads linking town and cities and the B roads are distributor roads with lower traffic density than A roads and are intended to connect different areas [34].

Built-environment variables included the number of schools, the number of bus stops, and the number of business establishments (as a proxy of activity levels) in each LTLA. School information was provided by the Department for Education “get information about schools” register (downloaded in December 2021 from https://get-information-schools.service.gov.uk/Downloads), bus stop locations were retrieved from Point of Interest Ordnance Survey data, and the number of business establishments were obtained from the Office for National Statistics. Note that some variables were available at a yearly basis. For other variables we considered the latest and/or the most relevant available data. This is because the data availability and LTLA boundaries have changed over the study period. The descriptive statistics of the explanatory variables and data sources and spatial distribution of a number of covariates are reported in Additional File 1.

### Statistical analysis

We used a Bayesian space-time Poisson lognormal model to evaluate associations between various relevant contributory factors and annual child pedestrian crashes, while accommodating dependencies between adjacent LTLAs and years. We initially attempted to account for spatio-temporal heterogeneity in the data through available covariates. However, due to unobserved/unmeasured factors that affect the outcome, which can be themselves spatially and temporally correlated, there remains residual autocorrelation. We dealt with the residual autocorrelation through specifying random effects that can account for the spatial and temporal dependencies and act as surrogate for unobserved/unmeasured covariates, affecting the outcome. This is achieved by including spatial, temporal, and spatio-temporal weight matrices that specify the neighbourhood structure, which allows us to exploit information sharing between neighbouring LTLAs and years.

After accounting for the covariates, we incorporated residual spatial variability through spatially structured random effects represented by a conditional autoregressive (CAR) prior [35]. The residual spatial term accounts for the dependencies between neighbouring LTLAs, which are conceptualised as LTLAs that share a common border. To account for the temporal dependency, we included temporally structured random effects using a random walk of order 1 (RW1), which captures the national temporal trend. Additionally, we included a space-time interaction term modelled as independent random walk for each LTLA [36]. The interaction term adds additional flexibility to the model and allows capturing local temporal deviations from the national (overall) time trend. Therefore, the temporal patterns in each LTLA are assumed to be temporally smooth but independent across space.

### Model specification

In our model specification, the number of observed crashes involving child pedestrians *y*_*it*_ in LTLA *i* (*i = 1, …,315)* and year *t (t = 1,..,10)* follows a Poisson distribution with the mean μ_it._ We then decompose the log (μ_it_) as

1 $$\log \left({\mu}_{it}\right)=\alpha +{X}_{it}\beta +{\phi}_i+{\xi}_t+{\delta}_{it}$$

where exp(α) represents the overall expected child pedestrian crash frequency (i.e., an intercept term), *ϕ*_*i*_ are spatially structured random effects that capture the main spatial patterns and ξ_t_ are temporally structured random effects to describe the global temporal pattern of crashes (i.e., national time trend) in England. The term δ_it_ represents a space-time interaction term and allows each LTLA to have a temporal trend deviating from the national one. The term **X**_it_ represents the covariate matrix of the *i*^*th*^ LTLA in time *t* and β are their respective regression coefficients. The spatially structured random effects *ϕ*_*i*_ are assigned a CAR prior as in

2 $${\phi}_i\mid {\phi}_{-i}\sim Normal\left(\frac{1}{n_i}\sum_{j\ne i}{\phi}_j,\frac{\sigma_{\phi}^2}{n_i}\right)$$

where *ϕ*_−*i*_ is the set of *ϕ*_*i*_ except for the *i*^*th*^ LTLA, *n*_*i*_ is the number of adjacent neighbours sharing a boundary to area *i,* and ${\sigma}_{\phi}^2$ is the variance of spatially structured random effects. The temporally structured random effects *ξ*_*t*_ are modelled using a random walk of order 1 (RW1) and is described as

3 $${\xi}_t\mid {\xi}_{t-1}\sim Normal\left({\xi}^{t-1},{\sigma}_{\xi}^2\right)$$

where ${\sigma}_{\xi}^2$ is the variance of temporal effects. In addition to the main temporal effects, we were interested in identifying localised departures. This was achieved by including a space-time interaction structure *δ*_*it*_ defined as

4 $${\delta}_{it}\sim Normal\left({\delta}^{t-1},{\sigma}_{\delta}^2\right)$$

As for priors, we assigned a Gamma (0.5, 0.005) to the inverse of variances ${\sigma}_{\phi}^2$, ${\sigma}_{\xi}^2$, and ${\sigma}_{\delta}^2$, a flat prior uniform(−∞,+∞) to *α*, and a Normal (0,1000) to the regression coefficients *β*. All explanatory variables were centred to improve the convergence of the model. For better interpretability of the explanatory variables, we obtained the magnitude of the effects of various covariates on child pedestrian safety in terms marginal effects [37] given by

5 $$\frac{\partial E\left(y|x\right)}{\partial {x}_p}=E\left(y|x\right){\beta}_p=\frac{1}{N}\sum_{i=1}^N\exp \left(\alpha +{X}_{it}\beta +{\phi}_i+{\xi}_t+{\delta}_{it}\right){\beta}_{i,p}$$

where subscript *p* refers to explanatory variables, here *p* = 6, and N is the number of observations.

Inferences were performed through Markov Chain Monte Carlo (MCMC) simulations in the NIMBLE Package in R [38]. We checked the convergence of the parameters using the Gelman-Rubin statistic [39] and visually using trace plots. In total, 20,000 post burn-in samples were obtained from the posterior distribution of the model parameters. In addition to the above-described model, we fitted other competing models with different specifications for space, time, and space-time effects, and compared the model fit using the Watanabe–Akaike information criterion (WAIC). However, these models did not improve the fit. Details of the competing models and their WAIC are reported in additional file 2.

We report the posterior summary of the magnitude of the effects of various covariates on child pedestrian safety in terms of marginal effects [40]. Marginal effects provide a more straightforward interpretation of the effect of covariates on safety, revealing the change in expected (mean) child pedestrian crashes following one unit change in each covariate. To check the goodness of fit of the model, we conducted posterior predictive checks and estimated Bayesian *p*-values [41], which is based on quantifying the discrepancies between predicted data, using the proposed model, and the observed data.

### Identifying high-crash areas (hotspots), spatial distribution of residuals and area-specific time trends

Using the expected crash frequency, we identified high-crash areas, where safety improvement programmes are most warranted. We classified LTLAs based on the posterior probability of the exponential of the spatial residuals in each LTLA being above one. If a probability was larger than 0.9 for an LTLA, it was classified as an LTLA with excess child pedestrian crash frequency (after accounting for explanatory variables that are in the model). Such areas are where other unknown/unmeasured risk factors (other than those in the model) have a negative impact on child pedestrian safety. Therefore, further investigation is needed to identify the reasons behind this, which in turn helps improve safety in those areas.

The inclusion of the space-time interaction term allowed each LTLA to have its own specific temporal trend, which is composed of the sum of the national temporal trend and the space-time interaction term. Similar to the approach adopted by Boulieri et al. [42], we report the probability that the estimated incidence of child pedestrian crashes in an LTLA represents an increase compared to the national one.

## Results

### LTLA factors affecting child pedestrian safety

Table 1 reports the estimated model parameters, including regression coefficients and parameters relating to our space-time interaction specification. We retained only statistically important covariates, considering those that were not highly correlated with each other, and that improved model fit. Child population, unemployment-related claimants, road density, and the number of schools were found to be positively associated with child pedestrian crash frequencies. However, licensed vehicles per capita and levels of walking and cycling were negatively associated with child pedestrian crash frequencies. With respect to model performance, we estimated Bayesian *p*-values for checking model adequacy. These being satisfactory are reported in the Additional File 3.

**Table 1:** Posterior summary of regression parameters

Statistically important explanatory variables	Mean	95% credible interval	
		2∙5%	97∙5%
Child population	0∙11	0∙08	0∙14
Unemployment-related claimants	0∙03	0∙01	0∙05
Licensed vehicles per capita	− 0∙55	− 0∙83	− 0∙26
Road density	0∙69	0∙52	0∙87
Adults who walk/cycle 3 times per week	−0∙01	− 0∙02	− 0∙002
Number of schools	0∙08	0∙07	0∙09
Model parameters			
exp(α)	11∙22	11∙09	11∙35
${\sigma}_{\phi}^2$ (Variance of structured spatial effect)	0∙535	0∙446	0∙644
${\sigma}_{\xi}^2$ (Variance of structured temporal effect)	0∙022	0∙010	0∙066
${\sigma}_{\delta}^2$ (Variance of the interaction term)	0∙004	0∙003	0∙005

To interpret the regression coefficients, Table 2 reports the magnitude of the impact of explanatory variables on child pedestrian crash frequencies in terms of marginal effects, indicating the magnitude of change in child crash frequency due to one unit change in an explanatory variable.

**Table 2:** Posterior summary of marginal effects

Statistically important explanatory variables	Mean (sd)	95% credible interval	
		2.5%	97.5%
Child population	1.80 (0.22)	1.35	2.23
Unemployment-related claimants	0.48 (0.17)	0.16	0.81
Licensed vehicles per capita	− 8.98 (2.34)	−13.35	−4.18
Road density	11.01 (1.47)	8.45	13.91
Adults who walk/cycle 3 times per week	−0.18 (0.08)	− 0.34	−0.04
Number of schools	1.27 (0.07)	1.11	1.42

**Fig. 1:**
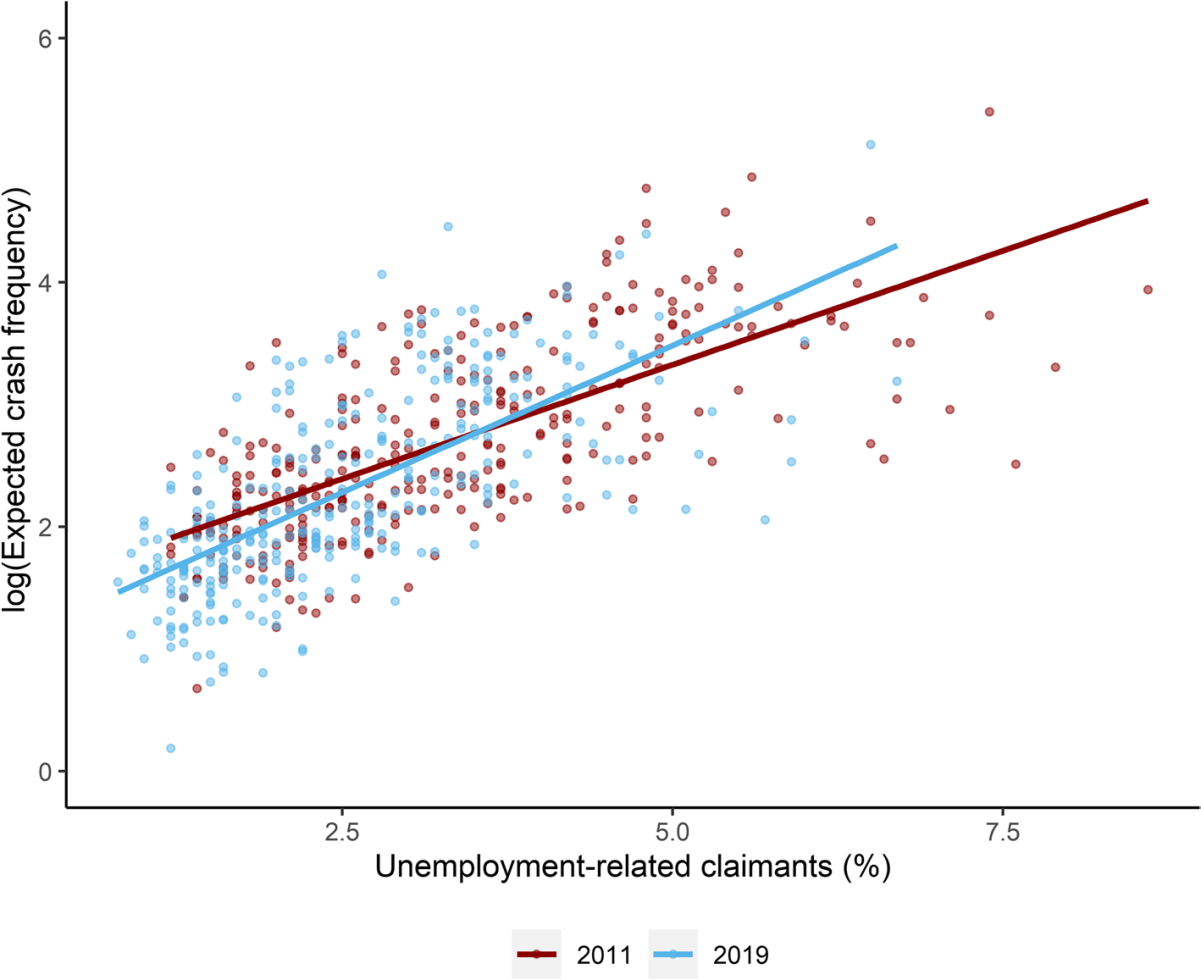
Expected crash frequency in relation to deprivation

In relation to deprivation, Fig. 1 displays that number of child pedestrian crashes (on log scale) in 2011 and 2019 in relation to the percent of population claiming some sort of unemployment-related benefit. Since 2020 included lock down periods and major shift in travel behaviour due to the COVID-19 pandemic [43, 44], we restricted our comparison to 2011 and 2019 as the two extremes of the study period. As shown in Fig. 1, the expected number of crashes was positively associated with the deprivation level. The slope of the 2019 line, however, is slightly larger than that of 2011, suggesting that as the percentage of unemployment-related claimants increases, crash frequencies increase at a higher rate in 2019 than in 2011. This implies socio-economic inequalities in child pedestrian safety increased in 2019 compared to 2011, highlighting the importance of the need for addressing inequity issues in this context.

### High-crash locations

**Fig. 2:**
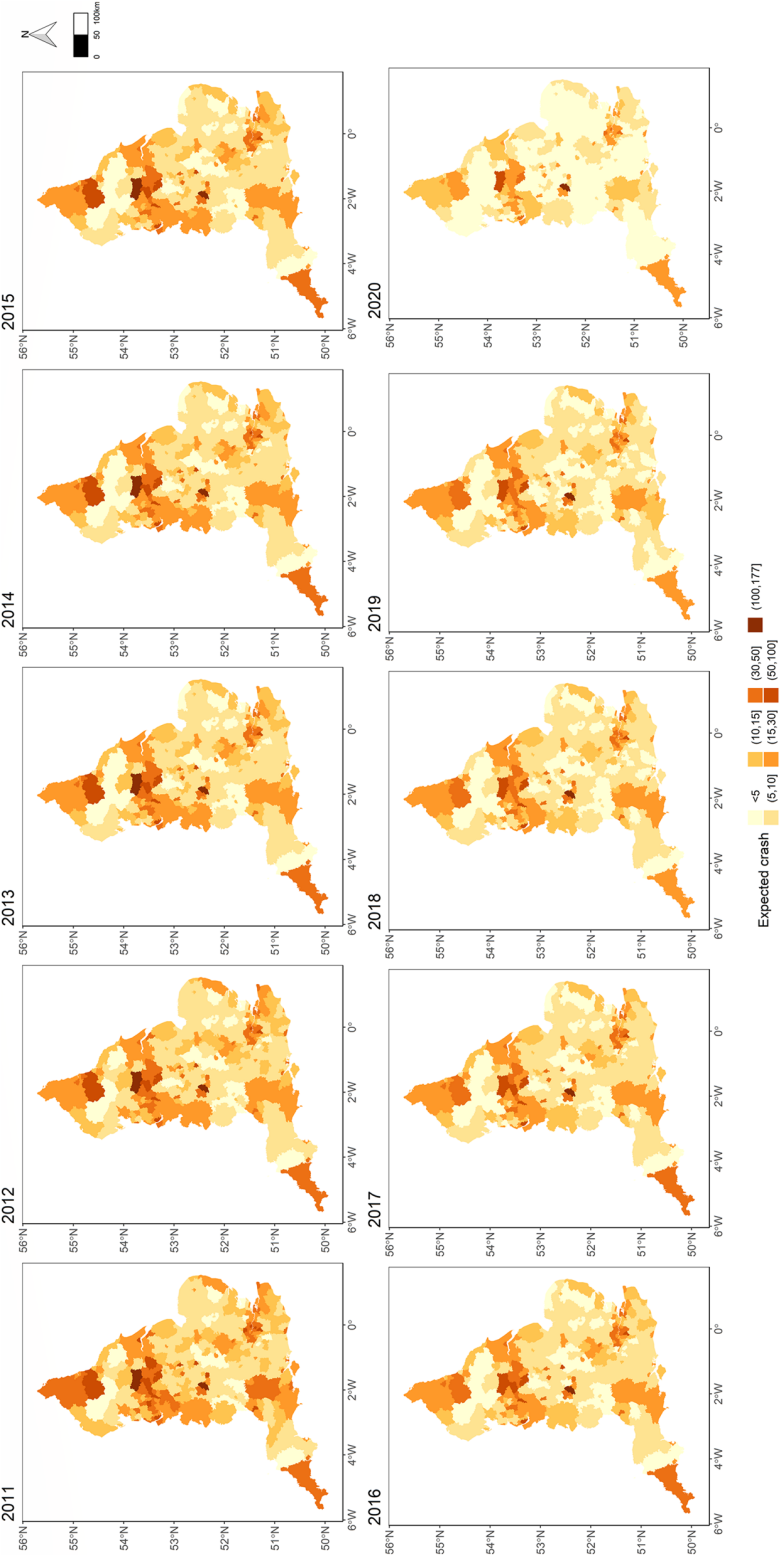
Spatial distribution of expected child pedestrian crash frequencies from 2011 to 2020

Figure 2 displays the spatial distribution of yearly expected child pedestrian crashes over the study period (the darker the colour, the higher the expected value). There is a relatively considerable spatial variation in expected child pedestrian crashes across England which remains visible across the years while we can also see the temporal evolution of child pedestrian safety in England over the last decade. Although we expect that urban areas have higher expected crash values, Fig. 2 can be used by local authorities to prioritise safety interventions and to inform resource allocation across England. For example, in 2019, Birmingham had the highest expected child pedestrian crash, followed by Leeds, Bradford, Liverpool, and Croydon, which all are characterized by low socioeconomic status.

### Overall spatial and temporal effects

**Fig. 3:**
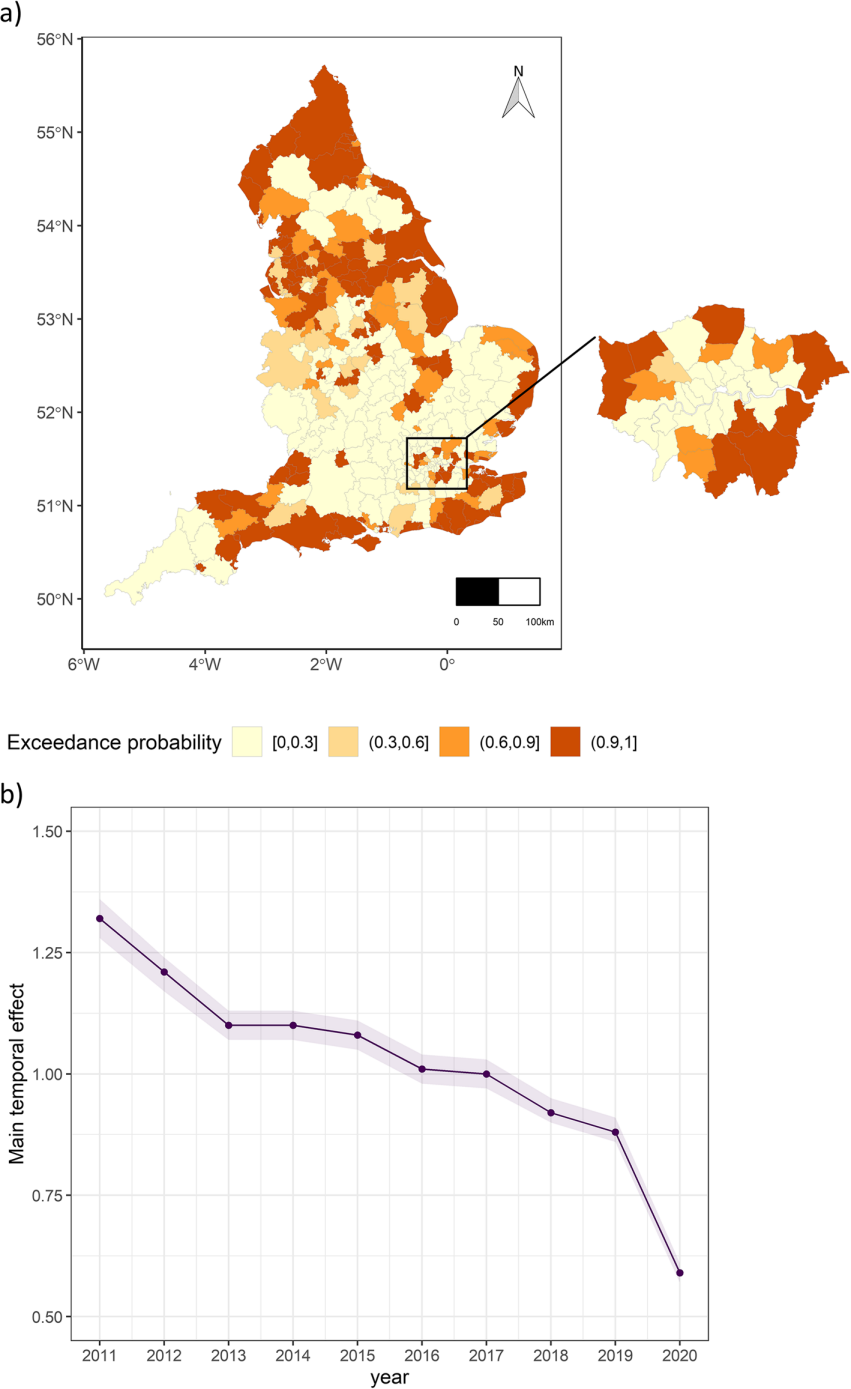
**a** Mapping posterior probability of spatial residuals being larger than 1. The map of Greater London is enlarged for better visualization. **b** Posterior median and 95% credible intervals of the overall (national) temporal trend

Figure 3a shows the map of exceedance probabilities of spatial residuals being greater than 1. This allows us to identify LTLAs with excess child pedestrian crash (shown in darker colour where the probability of exceedance is > 90%), after adjusting for the effect of the covariates. We found that 36% of the LTLAs (114 LTLAs) experienced excess crash from 2011 to 2020. These are mainly located in urban areas, especially in Northern England: Yorkshire and the Humber regions.

**Fig. 4:**
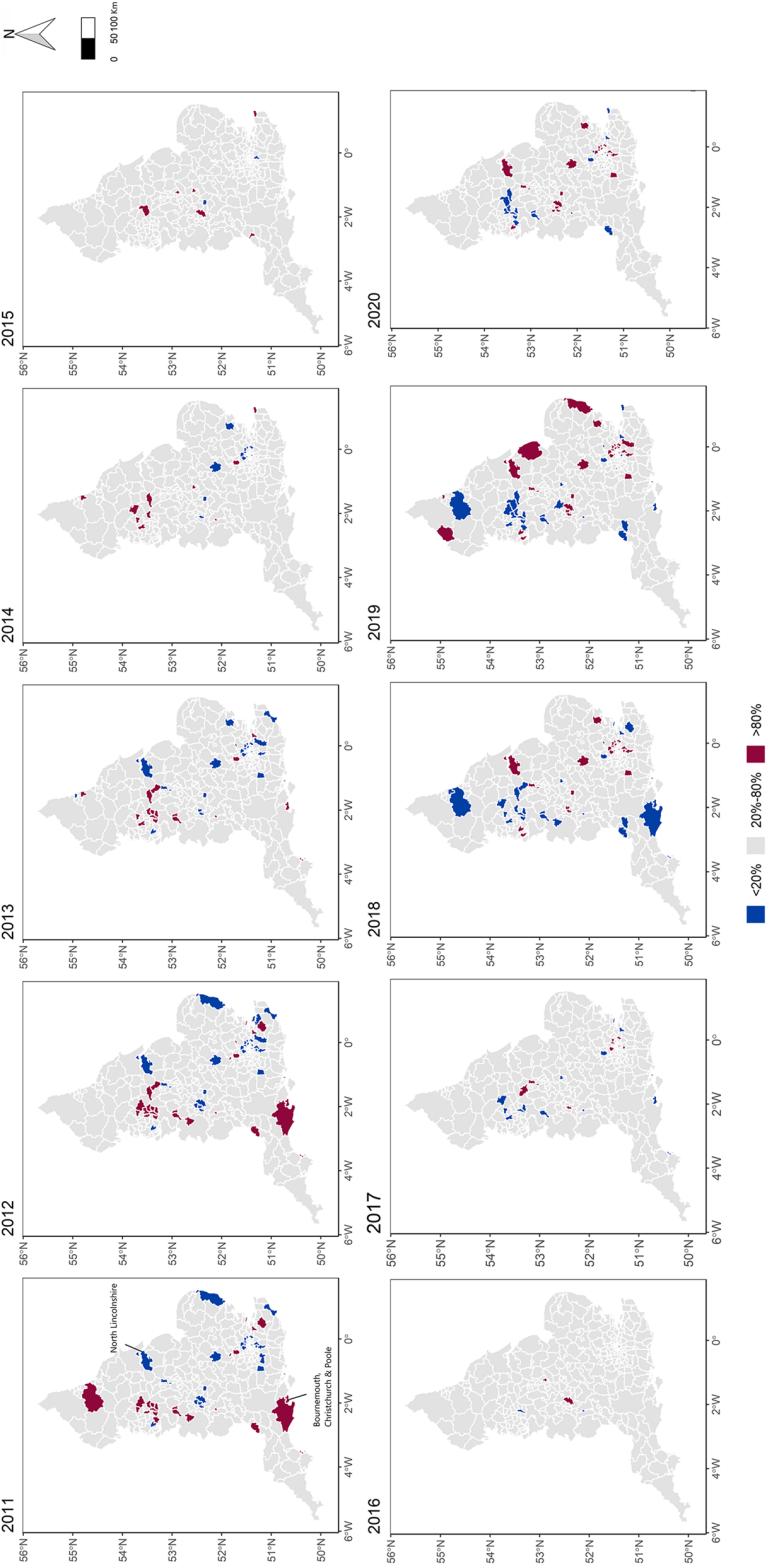
Probability of LTLA- specific time trend exceeding the national trend over the study period

Figure 3b shows the posterior median, including the 95% uncertainty band of the temporal trend, over the study period, representing the average national time trend. We observed a decreasing time trend, with year 2020 showing a much steeper decline compared to the other years, perhaps reflecting the effect of the Covid19 pandemic and its associated lockdown and work from home policies in England. Note that such policies resulted in reduced exposure (traffic volume, and walking and cycling) in general [45]. Between the years 2013 and 2017, we observed a relatively moderate but consistent decreasing trend. Figure 4 confirms a non-linear behaviour of temporal patterns over time.

### LTLA-specific time trends through space-time interaction

By specifying a space-time interaction term as an independent random walk for each LTLA, we were able to capture local time trends. Such local trends are due to the fact that the effects of some unknown highly localised variables vary smoothly over time while operating independently with respect to their locations. In fact, previous research in the field of road safety indicates that the effect of contributory factors may vary over time [46]. In the presence of missing localised variables (e.g., climate), the interaction term can act as a surrogate measure for these unmeasured/unknown variables. This allows us to capture their effects to some extent, thereby addressing unobserved heterogeneity more fully. The time trend for all LTLAs exhibited downward trend (similar to the national trend shown in Fig. 3b) with different degrees of deviation from the national trend (see Additional File 4). The decline in crash incidence was slower in some local authorities when compared to the national trend.

Figure 4 displays the map of the probability that, after accounting for the covariates, the incidence of child pedestrian crashes is higher than the national one in each LTLA in each year. The incidence of child pedestrian crash in North Lincolnshire was below the average national incidence between 2011 and 2013, but it exceeded the national value (with probability > 80%) after 2018. This trend suggests that there are specific risk factors in North Lincolnshire that contribute to the deterioration of road safety for children, which requires further in-depth investigations. In contrast, the crash incidence in the local authority of Bournemouth, Christchurch and Poole in 2011–2013 resulted higher than the average national, but child pedestrian safety improved over the recent years such that after 2017 the incidence of crash became less than the average national child pedestrian crash incidence. For a detailed visualisation, the time trend for the highlighted local authorities (shown blue and red colours) in comparison to the national trend is illustrated in the Additional File 4.

## Discussions

### Association between LTLAs characteristics and child pedestrian safety

Identifying contributory factors affecting zonal-level child pedestrian safety can provide useful insights toward designing and implementing effective large-scale countermeasures. For example, based on our findings, increased levels of walking and cycling helps increase road safety for children. This is an interesting finding that, in accordance with previous research (see; e.g., Stoker et al., [47] and Jacobsen et al., [48]) indicates that the higher the prevalence of walking and cycling in LTLAs, the safer the road network for child pedestrians. Also, this could be attributed to motorists adjusting their driving behaviours (e.g., by lowering driving speeds) in the presence of increased numbers of pedestrians and cyclists [49–51].

Specifically, based on marginal effects reported in the section of results, we found that one unit increase in road density can lead to an additional 11.01 child pedestrian crashes annually. Road density can act as a proxy exposure measure for motorised traffic, which is known to have a deteriorating effect on pedestrian safety [52]. Our finding regarding road density is consistent with the results of previous studies [53]. One unit increase in child population, on average, resulted in 1.8 additional child pedestrian crashes per year. For every 10 additional schools, expected child pedestrian crash frequencies increased by 1.27 per year. One potential explanation is that an increase in child population and the number of schools leads to an increase in exposure [26, 49, 54]. In terms of deprivation, one unit (here, 1 %) increase in unemployment-related claimants resulted in 0.48 additional child pedestrian crashes per year. In contrast, one unit increase in the number of licensed vehicles per capita decreased child pedestrian crash frequencies by 8.98 crashes per year. One explanation for this finding could be that children in areas with higher number of licensed vehicles are more likely to travel by car rather than walking, reducing their exposure. Also, the latter two variables often relate to deprivation and previous studies have also found similar results [15, 26]. Finally, 1 % increase of adults who walk or cycle at least three times per week decreased expected child pedestrian crash frequencies by 0.18 crashes per year.

One important factor is deprivation which has a negative impact on road safety. The socio-economic disparities in child pedestrian crashes might be partially driven by exposure disparities as children in deprived areas are more likely to walk to school [55, 56] Therefore, road safety policies need to target more deprived areas through safety improvement programmes such as reducing traffic volume and speed, designing walking-friendly infrastructures, education, and training programmes. In addition, whilst child pedestrian crash frequencies have declined over the last decade, its association with deprivation over time has not changed substantially. If road safety interventions successfully target deprived areas, the association between deprivation and child crash frequency would weaken in the future. With increasing interest in policies to encourage children to walk, efforts to improve child pedestrian safety is successful only when a system approach is adopted, emphasising on data-informed engineering interventions in conjunction with interventions that address deprivation.

### Spatio-temporal variations in child pedestrian safety

Since 2011, child pedestrian crashes have decreased by more than 50% in England. However, some local authorities still struggle to improve road safety conditions for children. Overall, the southern part of England has experienced higher levels of improvement in child pedestrian safety over the last decade compared to the northern regions. Local authorities of Birmingham, Leeds, and Bradford had consistently the highest expected child pedestrian crash frequency throughout England over the study period. In 2011, there was a difference of 218.3 [95% CrI 197.1–240.9] crashes between the LTLA with the lowest (Rutland) and the highest child pedestrian crash frequencies (Birmingham). Although road safety has in general improved over the study period, in 2020, there was a gap of 112.22 [98.9–125.4] expected annual crashes between LTLAs (Rutland and Birmingham) with the highest and the lowest expected crash frequencies.

Estimating the LTLA-specific time trends suggests the presence of localised risk factors or may reflect the impact of local interventions and policies, which requires further in-depth investigations. This can be particularly important from a public health perspective and for implementing cost-effective safety interventions. We noticed a major reduction in expected child pedestrian crash frequencies in most local authorities in 2020, which is expected due to the recent pandemic (see, for example, the work by Katrakazas et al. [57] for a discussion on the effect of the Covid-19 pandemic on road safety). However, the expected child pedestrian crash frequency was less affected in certain regions such as Cornwall and Northumberland.

From a policy insight perspective and with the aim of improving child pedestrian safety across England, our results can be used to prioritise safety interventions and to inform resource allocation across England.. This can be achieved based on the identification of high-crash local authorities (so called hotspots), understanding factors affecting child pedestrian safety, and tracking how safety conditions, in terms of child pedestrian safety, at different local authorities have evolved over the study period.

### Strengths and limitations of the study

We accommodated spatial dependencies that could potentially account for similarities in unobservable factors and travel patterns (e.g., children mobility and traffic volume) in neighbouring local authorities. Our specification of space-time interaction allowed each local authority to have its own temporal pattern. Consequently, this specification not only led to more reliable statistical inferences but also allowed us to provide further insights with the same set of data.

A limitation of our study is that we could not use some other potentially useful variables such as traffic volume and land use characteristics in our analysis as their LTLA boundaries did not match the boundaries associated with our outcome of interest. However, we showed that our model performs very well in replicating the observed data so this would not cause any major issue in this study. This is partly because we indirectly accounted for these omitted variables in the model. For example, we included road density as a proxy measure for traffic volume. Due to lack of data availability, we removed Isles of Scilly and City of London. However, since the number of observed crashes in these two local authorities was very small, we do not expect that their removal substantially affects the results.

## Conclusions

To our knowledge, this study is the first to explore spatial-temporal patterns of child pedestrian crashes at local authority level in England over a long-time span,10 years (2011–2020). We used a Bayesian spatio-temporal model where we included relevant covariates, accounted for spatial and temporal dependencies in the data as well as allowing for a spatio-temporal interaction. This enabled us to (i) identify statistically important area-level variables that can explain child pedestrian safety, (ii) reveal spatial patterns and national trend in child pedestrian crashes across England over the last decade, and (iii) understand how road safety conditions evolved in each local authority over the study period. The results indicate that child pedestrian crashes have been gradually declining in England over the last decade. Some local authorities (mainly in urban areas of northern England) exhibited higher child crash frequencies than national average over the study period. More deprived local authorities have been experiencing a higher number of child pedestrian crashes and there is no evidence suggesting that socioeconomic-related inequality gap has narrowed from 2011. Efforts to improve child pedestrian safety would be more successful if emphasise is given to areas where safety improvements are most warranted and to evidence-based policy making in conjunction with interventions that can address social inequalities.

## Supplementary Information

**Additional file 1: **information on data. **Figure 1.** Geocoded location of child pedestrian crashes across England from 2011 to 2020. **Table 1.** Summary statistics of the explanatory variables. **Figure 2.** Spatial distribution of road density in England (1/km). **Figure 3.** Spatial distribution of Number of schools in England. **Figure 4.** Spatial distribution of the number of vehicles per capita in England. **Figure 5.** A Spatial distribution of the percent of adults who walk/cycle at least three times per week in England. **Figure 6.** Spatial distribution of the percent of child population over years in England. **Figure 7.** Spatial distribution of the percent of population who claim unemployment-related benefit over years in England.

**Additional file 2: **Competing models and model comparison. **Table 2.** Specification of competing models. **Table 3.** The model fit and the estimated parameters with 95% credible intervals for competing models.

**Additional file 3: **Checking goodness-of-fit. **Table 4.** Average Bayesian *p*-values.

**Additional file 4: **LTLA-specific time trends. **Figure 8.** LTLA-specific time trends. **Figure 9.** Time trend of local authorities where the crash incidence exceeds the average national value over time. **Figure 10.** Time trend of local authorities where the crash incidence goes below the average national value over time.

**Additional file 5.**

## Data Availability

All data generated or analysed during this study are included in this published article and its supplementary information files (Additional file 5).

## Abbreviations

LTLA: lower tier local authority
CAR: conditional autoregressive
RW1: random walk of order 1
MCMC: Markov Chain Monte Carlo
WAIC: Watanabe–Akaike information criterion
