# Supplementary material for: A decade of child pedestrian safety in England: a bayesian spatio-temporal analysis
Source: BMC Public Health. 2023 Feb 1;23:215. doi: 10.1186/s12889-023-15110-2 (PMC9889245; doi:10.1186/s12889-023-15110-2)
Supplement: Supplementary file 1 — Additional file 1: information on data. Figure 1. Geocoded location of child pedestrian crashes across England from 2011 to 2020. Table 1. Summary statistics of the explanatory variables. Figure 2. Spatial distribution of road density in England (1/km). Figure 3. Spatial distribution of Number of schools in England. Figure 4. Spatial distribution of the number of vehicles per capita in England. Figure 5. A Spatial distribution of the percent of adults who walk/cycle at least three times per week in England. Figure 6. Spatial distribution of the percent of child population over years in England. Figure 7. Spatial distribution of the percent of population who claim unemployment-related benefit over years in England. [file 12889_2023_15110_MOESM1_ESM.docx]

# Additional file 1: information on data


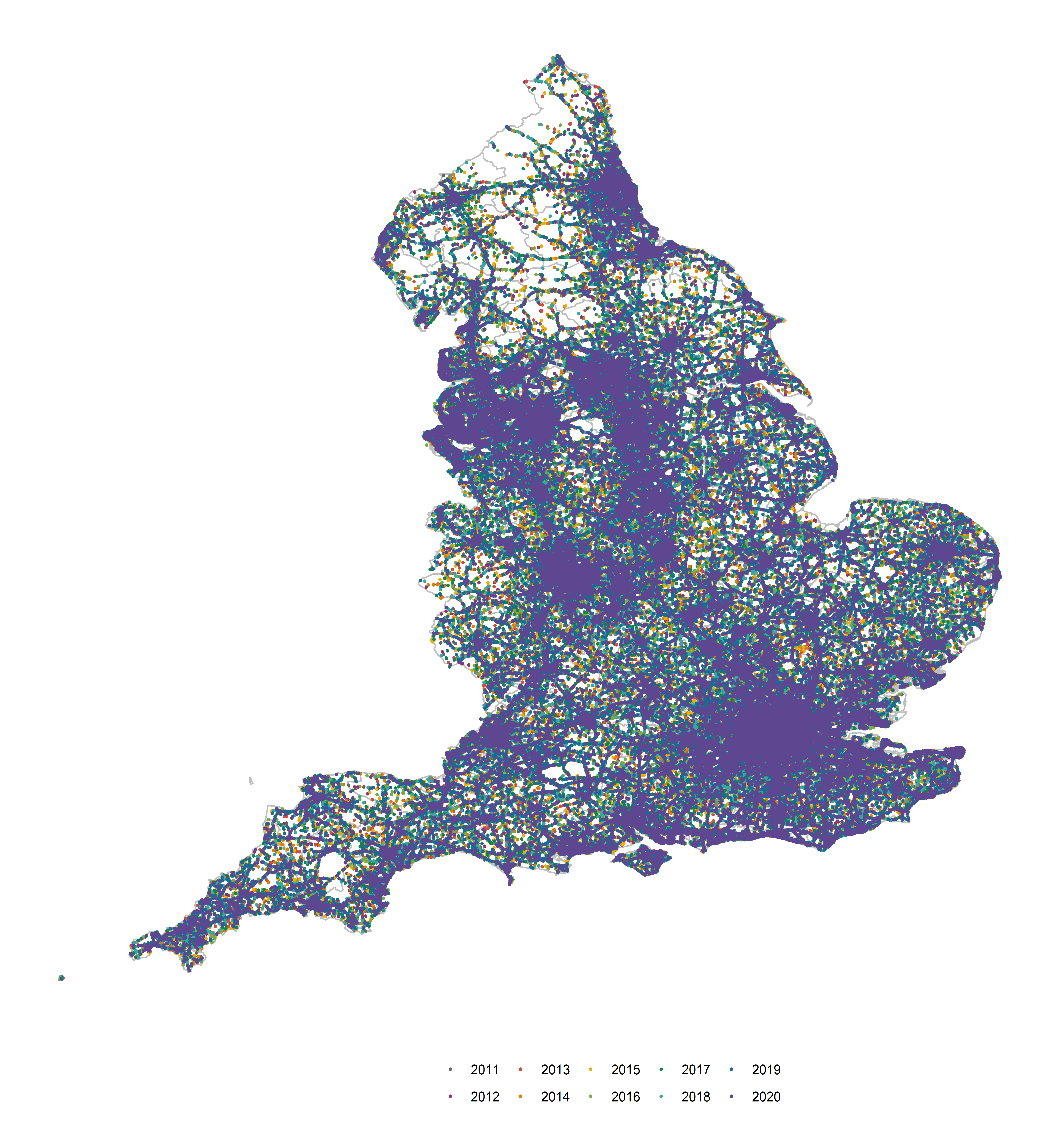


Figure 1. Geocoded location of child pedestrian crashes across England from 2011 to 2020

Table 1. Summary statistics of the explanatory variables

| **Variable** | **Data sources** | **Year** | **Mean** | **Standard deviation** | **Min** | **Max** |
| --- | --- | --- | --- | --- | --- | --- |
| *Socio-demographic variables* | | | | | | |
| Child population (%) | Office for National Statistics | 2011 | 18.63 | 1.74 | 14.40 | 25.90 |
|  |  | 2012 | 18.66 | 1.79 | 14.20 | 26.40 |
|  |  | 2013 | 18.65 | 1.85 | 14.00 | 26.80 |
|  |  | 2014 | 18.64 | 1.89 | 14.00 | 26.90 |
|  |  | 2015 | 18.66 | 1.94 | 14.00 | 27.00 |
|  |  | 2016 | 18.70 | 1.98 | 14.00 | 27.20 |
|  |  | 2017 | 18.78 | 2.02 | 14.00 | 27.30 |
|  |  | 2018 | 18.85 | 2.06 | 13.90 | 27.30 |
|  |  | 2019 | 18.87 | 2.09 | 13.80 | 27.20 |
|  |  | 2020 | 18.85 | 2.13 | 13.60 | 27.30 |
| Unemployment rate | Office for National Statistics | 2011 | 7.66 | 3.10 | 1.80 | 22.30 |
|  |  | 2012 | 7.52 | 2.93 | 2.10 | 16.50 |
|  |  | 2013 | 6.96 | 2.98 | 1.70 | 16.10 |
|  |  | 2014 | 6.05 | 2.45 | 2.00 | 14.70 |
|  |  | 2015 | 5.15 | 2.06 | 1.60 | 12.10 |
|  |  | 2016 | 4.92 | 1.83 | 1.70 | 10.80 |
|  |  | 2017 | 4.63 | 1.83 | 1.30 | 11.50 |
|  |  | 2018 | 4.56 | 1.61 | 1.10 | 10.60 |
|  |  | 2019 | 4.42 | 1.60 | 1.10 | 9.20 |
|  |  | 2010 | 4.99 | 1.82 | 1.70 | 13.00 |
| Population who are managers, directors and senior official (%) | Office for National Statistics | 2011 | 10.60 | 3.50 | 2.40 | 24.80 |
|  |  | 2012 | 10.67 | 3.45 | 3.10 | 24.90 |
|  |  | 2013 | 10.73 | 3.57 | 2.50 | 24.80 |
|  |  | 2014 | 10.78 | 3.21 | 4.40 | 23.50 |
|  |  | 2015 | 10.99 | 3.60 | 3.00 | 27.70 |
|  |  | 2016 | 11.15 | 3.68 | 2.60 | 27.00 |
|  |  | 2017 | 11.71 | 4.07 | 3.10 | 25.80 |
|  |  | 2018 | 11.52 | 3.92 | 4.00 | 28.20 |
|  |  | 2019 | 12.13 | 4.20 | 3.40 | 34.00 |
|  |  | 2010 | 12.09 | 4.14 | 4.50 | 33.00 |
| Job density | Office for National Statistics | 2011 | 0.77 | 0.26 | 0.39 | 4.16 |
|  |  | 2012 | 0.78 | 0.27 | 0.40 | 4.26 |
|  |  | 2013 | 0.79 | 0.27 | 0.41 | 4.39 |
|  |  | 2014 | 0.80 | 0.28 | 0.43 | 4.45 |
|  |  | 2015 | 0.82 | 0.28 | 0.40 | 4.42 |
|  |  | 2016 | 0.84 | 0.28 | 0.40 | 4.42 |
|  |  | 2017 | 0.85 | 0.28 | 0.42 | 4.33 |
|  |  | 2018 | 0.85 | 0.28 | 0.39 | 4.29 |
|  |  | 2019 | 0.86 | 0.28 | 0.40 | 4.35 |
|  |  | 2020 | 0.83 | 0.26 | 0.39 | 3.93 |
| Unemployment-related claimants (%) | Office for National Statistics | 2011 | 3.30 | 1.47 | 1.20 | 8.60 |
|  |  | 2012 | 3.20 | 1.50 | 1.10 | 8.70 |
|  |  | 2013 | 2.47 | 1.25 | 0.80 | 6.90 |
|  |  | 2014 | 1.64 | 0.91 | 0.50 | 4.90 |
|  |  | 2015 | 1.45 | 0.83 | 0.40 | 4.60 |
|  |  | 2016 | 1.49 | 0.84 | 0.40 | 4.70 |
|  |  | 2017 | 1.64 | 0.94 | 0.50 | 6.60 |
|  |  | 2018 | 2.01 | 1.09 | 0.40 | 6.90 |
|  |  | 2019 | 2.54 | 1.14 | 0.80 | 6.70 |
|  |  | 2020 | 5.68 | 1.87 | 2.90 | 12.00 |
| Number of licensed vehicles per capita | Department for Transport | 2020 | 0.63 | 0.20 | 0.17 | 1.83 |
| White population (%) | Office for National Statistics | 2011 | 90.65 | 12.07 | 27.10 | 100 |
|  |  | 2012 | 90.83 | 12.00 | 29.70 | 100 |
|  |  | 2013 | 90.76 | 12.15 | 27.30 | 100 |
|  |  | 2014 | 90.44 | 12.06 | 32.60 | 100 |
|  |  | 2015 | 90.30 | 12.38 | 35.10 | 100 |
|  |  | 2016 | 90.04 | 12.39 | 38.10 | 100 |
|  |  | 2017 | 89.85 | 12.28 | 35.60 | 100 |
|  |  | 2018 | 89.60 | 12.48 | 34.10 | 100 |
|  |  | 2019 | 89.48 | 12.39 | 39.20 | 100 |
|  |  | 2020 | 89.56 | 12.22 | 38.10 | 100 |
| *Transport-related variables* | | | | | | |
| Adults who walk/cycle at least 3 times/ week (%) | Department for Transport | 2019 | 47.71 | 6.17 | 31.27 | 73.05 |
| Adults who walk/cycle at least 5 times/week (%) | Department for Transport | 2019 | 35.61 | 5.71 | 21.83 | 61.38 |
| Length of A roads (km) | Ordnance Survey Meridian | 2016 | 102.19 | 92.40 | 6.87 | 695.72 |
| Length of B roads (km) | Ordnance Survey Meridian | 2016 | 62.23 | 77.60 | 0.00 | 631.67 |
| Road density | Ordnance Survey Meridian | 2016 | 0.50 | 0.43 | 0.02 | 2.93 |
| *Built-environment variables* | | | | | | |
| Number of schools | Department for Education | 2020 | 77.46 | 48.55 | 20.00 | 501.00 |
| Number of bus stops | Ordnance Survey Point of Interest | 2015 | 936.90 | 697.95 | 174.00 | 4997.00 |
| Number of business establishments | Office for National Statistics | 2019 | 8606 | 6302.11 | 2165 | 58910 |

## Below are the maps of a number of covariates.


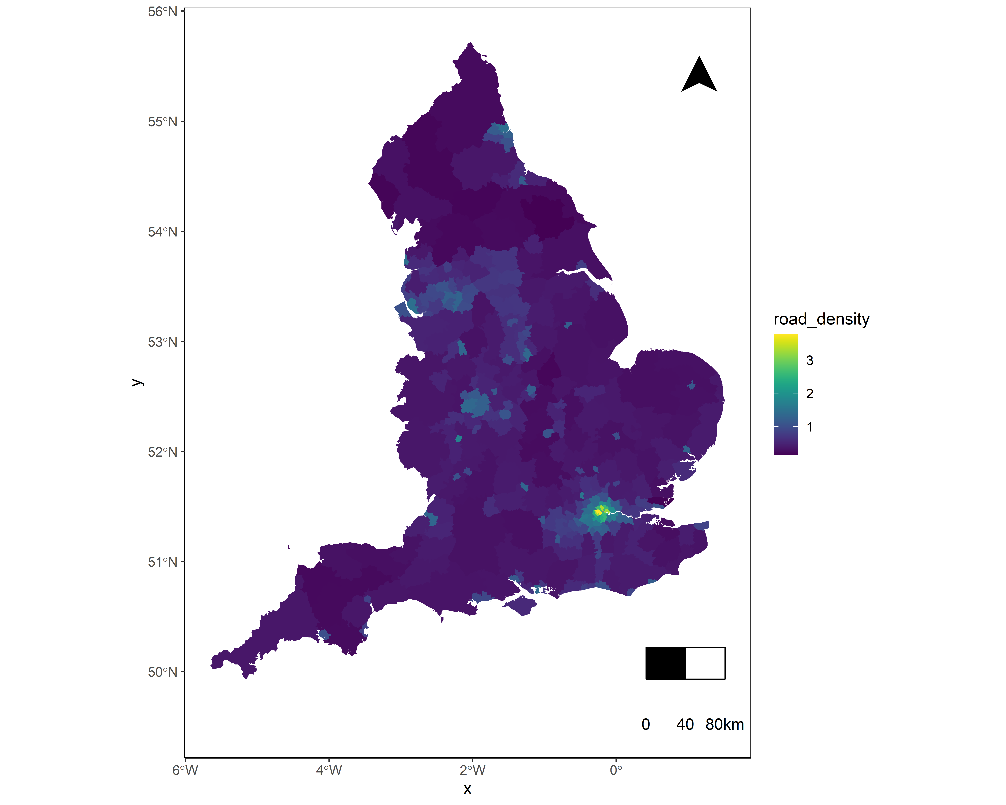


Figure 2 Spatial distribution of road density in England (1/km)


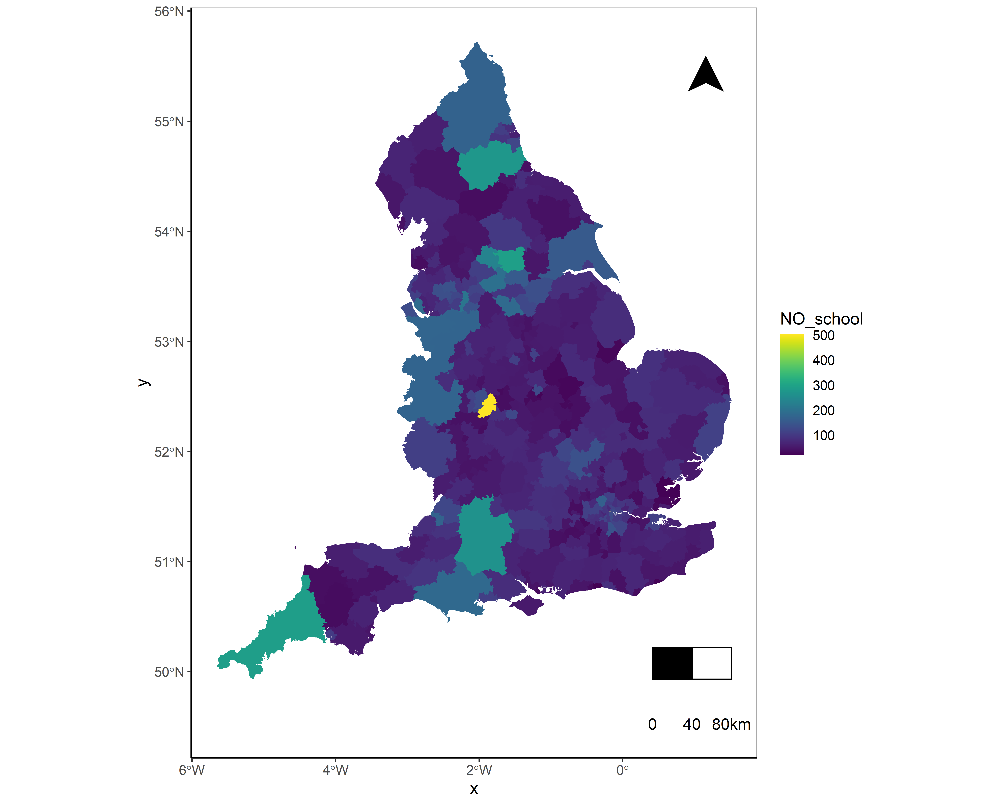


Figure 3 Spatial distribution of Number of schools in England


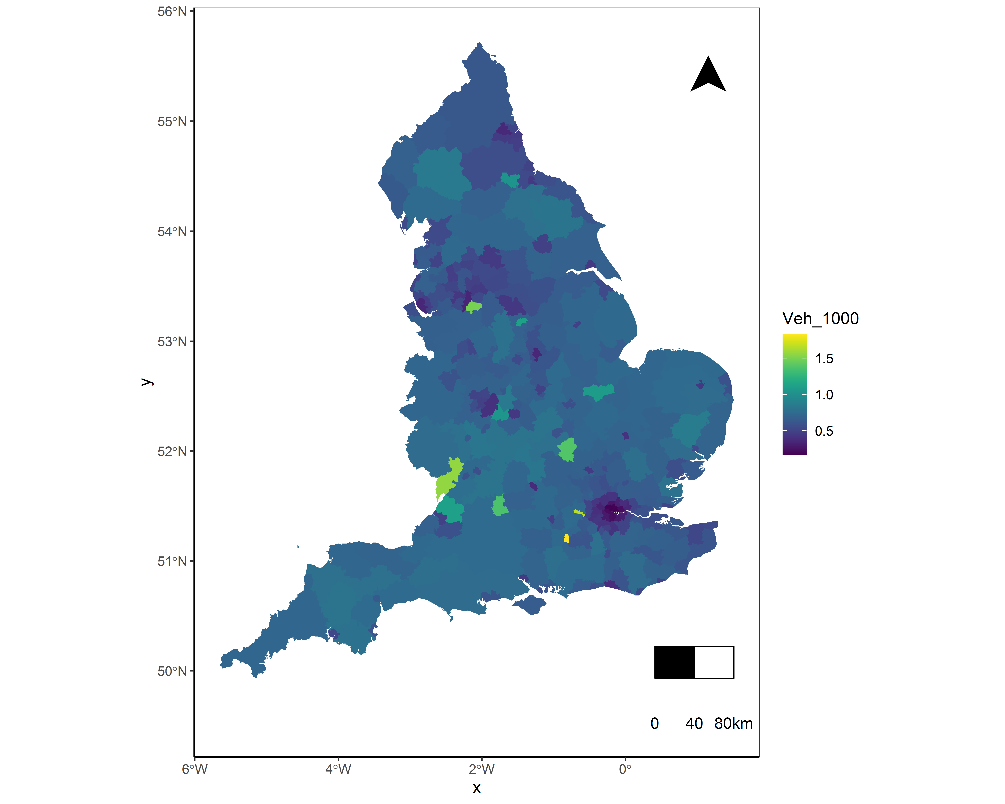


Figure 4 Spatial distribution of the number of vehicles per capita in England


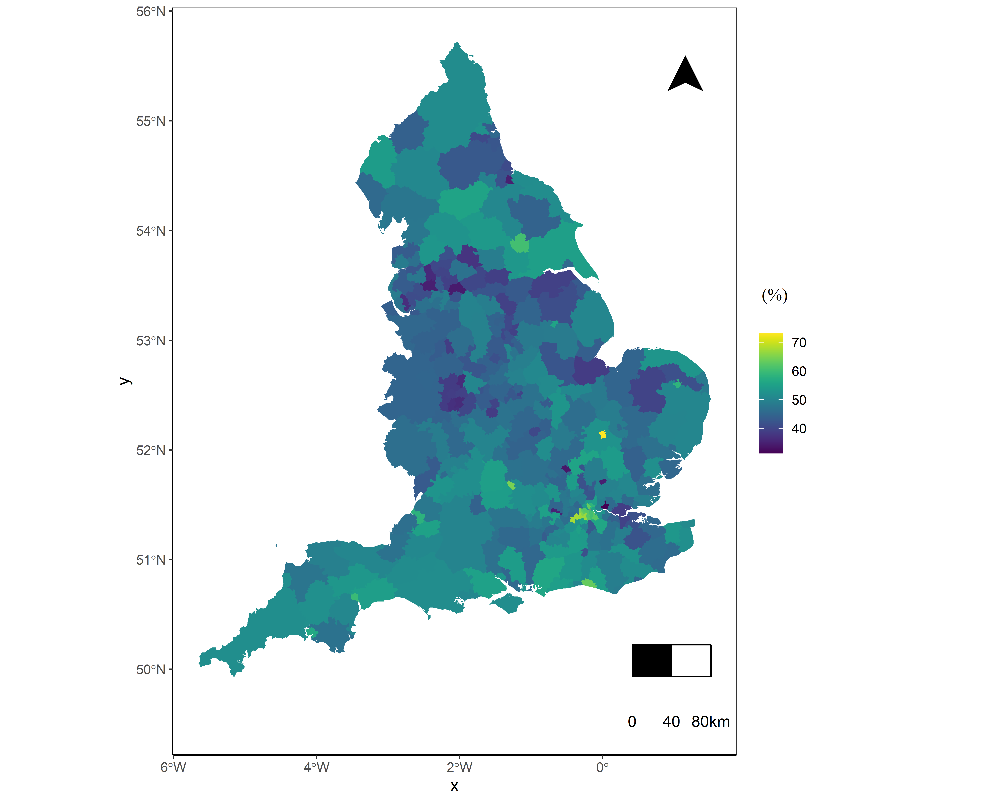


Figure 5 Spatial distribution of the percent of adults who walk/cycle at least three times per week in England


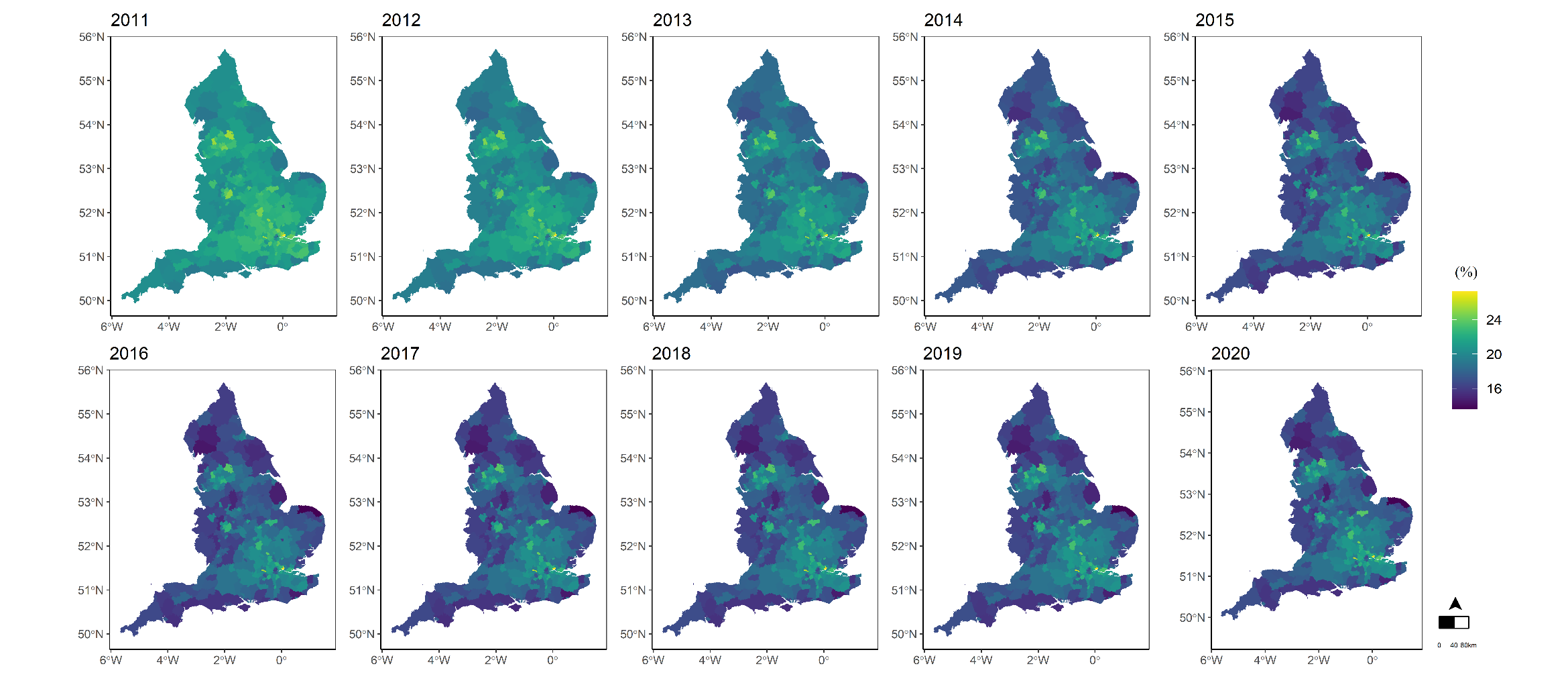


Figure 6 Spatial distribution of the percent of child population over years in England


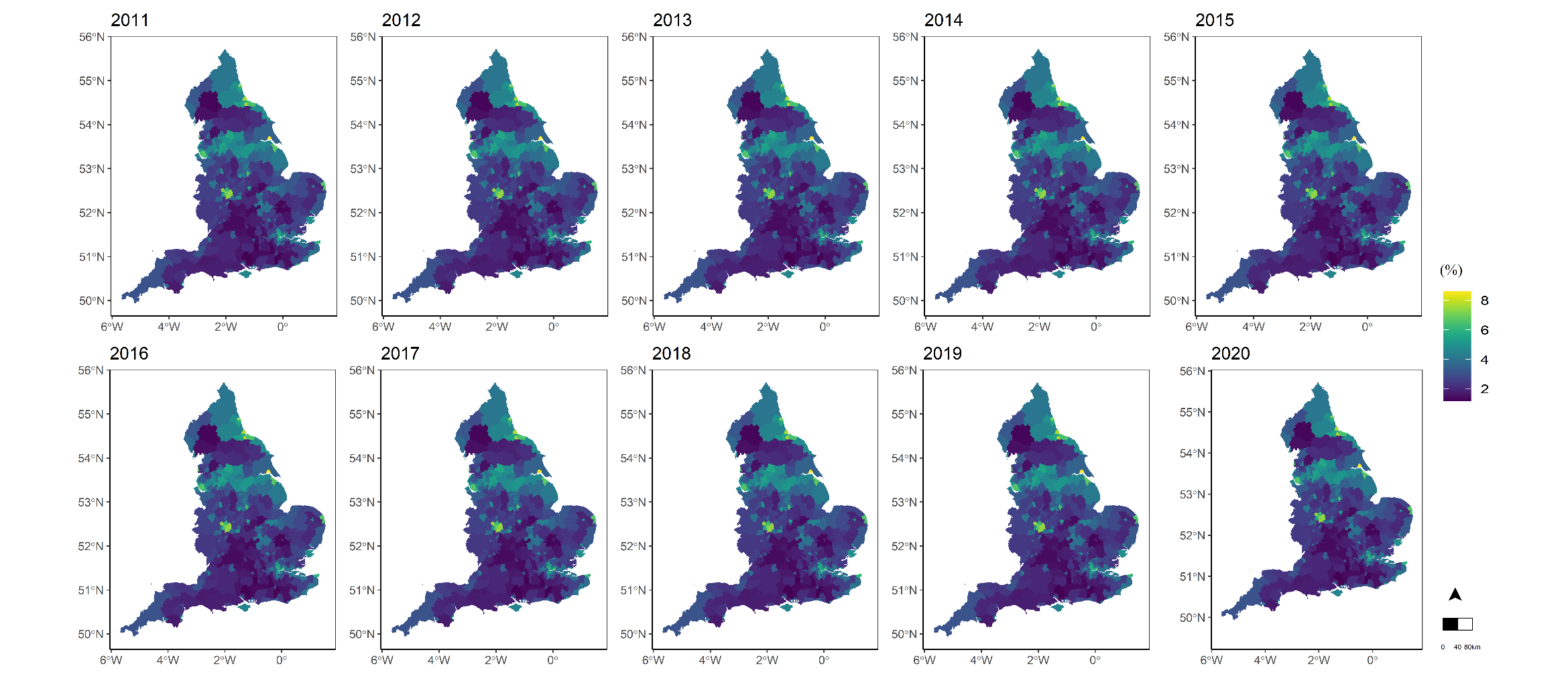


## Figure 7 Spatial distribution of the percent of population who claim unemployment-related benefit over years in England
