# Supplementary material for: A decade of child pedestrian safety in England: a bayesian spatio-temporal analysis
Source: BMC Public Health. 2023 Feb 1;23:215. doi: 10.1186/s12889-023-15110-2 (PMC9889245; doi:10.1186/s12889-023-15110-2)
Supplement: Supplementary file 2 — Additional file 2: Competing models and model comparison. Table 2. Specification of competing models. Table 3. The model fit and the estimated parameters with 95% credible intervals for competing models. [file 12889_2023_15110_MOESM2_ESM.docx]

## *Additional file 2: Competing models and model comparison*

We had also considered other specifications under various assumption regarding the spatial, temporal, and space-time interaction random effects. Model (1) included only spatial trend within the framework of the Besag, York, and Mollié (BYM), where information is shared both locally (among neighbouring LTLAs) through a spatially structured random effects modelled with CAR, and globally, through spatially unstructured Gaussian random effects. Models (2) and (3) additionally included a time-trend component, one through temporally structured random effects modelled with RW1 and the other one as unstructured random effect, modelled with a Gaussian prior. Model (4) included all components described in the previous three models. Model (5) focuses only on the spatially and temporally structured random effects. We then then included space-time interaction term in model (6) with no dependence structure, implying that the space-time residuals do not vary smoothly over space or time. Model (7) has additional flexibility compared to model (6) as it can capture localised time trends as we assume a RW1 structure for the interaction term. A summary of all competing models is reported in Table 2.

Table 2. Specification of competing models

| **Model** | **Model specification** | **Spatial component** | **Temporal component** | **Space-time interaction** |
| --- | --- | --- | --- | --- |
| 1 | $\alpha+\phi_{i}+\theta_{i}+X_{it}\beta$ | $\phi_{i}\sim CAR$  $\theta_{i}\sim normal$ | - | - |
| 2 | $\alpha+\phi_{i}+\theta_{i}+\xi_{t}+X_{it}\beta$ | $\phi_{i}\sim CAR$  $\theta_{i}\sim normal$ | $\xi_{t}\sim RW1$ | - |
| 3 | $\alpha+\phi_{i}+\theta_{i}+\tau_{t}+X_{it}\beta$ | $\phi_{i}\sim CAR$  $\theta_{i}\sim normal$ | $\tau_{t}\sim normal$ | - |
| 4 | $\alpha+\phi_{i}+\theta_{i}+\xi_{t}+{\tau_{t}+X}_{it}\beta$ | $\phi_{i}\sim CAR$  $\theta_{i}\sim normal$ | $\xi_{t}\sim RW1$  $\tau_{t}\sim normal$ | - |
| 5 | $\alpha+\phi_{i}+\xi_{t}+X_{it}\beta$ | $\phi_{i}\sim CAR$ | $\xi_{t}\sim RW1$ | - |
| 6 | $\alpha+\phi_{i}+\xi_{t}+\delta_{it}+X_{it}\beta$ | $\phi_{i}\sim CAR$ | $\xi_{t}\sim RW1$ | $\delta_{it}\sim normal$ |
| 7 | $\alpha+\phi_{i}+\xi_{t}+\delta_{it}+X_{it}\beta$ | $\phi_{i}\sim CAR$ | $\xi_{t}\sim RW1$ | $\delta_{it}\sim RW1$ |

Comparing the WAIC of different model, model 7 was selected as the best performing one and, consequently, was discussed in the paper. The WAIC values for competing models are reported in Table 3 of appendix.

Table 3. The model fit and the estimated parameters with 95% credible intervals for competing models

| **Model** | **WAIC** | $\boldsymbol{\sigma}_{\boldsymbol{\phi}}^{\boldsymbol{2}}$ | $\boldsymbol{\sigma}_{\boldsymbol{\theta}}^{\boldsymbol{2}}$ | $\boldsymbol{\sigma}_{\boldsymbol{\zeta}}^{\boldsymbol{2}}$ | $\boldsymbol{\sigma}_{\boldsymbol{\tau}}^{\boldsymbol{2}}$ | $\boldsymbol{\sigma}_{\boldsymbol{\delta}}^{\boldsymbol{2}}$ |
| --- | --- | --- | --- | --- | --- | --- |
| 1 | 19042 | 0∙10  [0∙03-0∙22] | 0∙11  [0∙08-0∙15] | - | - | - |
| 2 | 17144 | 0∙12  [0∙05-0∙22] | 0∙09  [0∙06-0∙12] | 0∙03  [0∙01-0∙06] | - | - |
| 3 | 17143 | 0∙12  [0∙05-0∙21] | 0∙05  [0∙02-0∙13] | - | 0∙09  [0∙06-0∙12] | - |
| 4 | 17143 | 0∙12  [0∙05-0∙22] | 0∙09  [0∙06-0∙12] | 0∙02  [0∙01-0∙06] | 0∙09  [0∙06-0∙12] | - |
| 5 | 17146 | 0∙54  [0∙45-0∙65] | - | 0∙03  [0∙01-0∙06] | - | - |
| 6 | 17094 | 0∙54  [0∙45-0∙64] | - | 0∙03  [0∙01-0∙06] | - | 0∙005  [0∙002-0∙006] |
| **7** | **17020** | **0∙54**  **[0∙45-0∙64]** | **-** | **0∙03**  **[0∙01-0∙07]** | **-** | **0∙003**  **[0∙002-0∙005]** |
