# Supplementary material for: A decade of child pedestrian safety in England: a bayesian spatio-temporal analysis
Source: BMC Public Health. 2023 Feb 1;23:215. doi: 10.1186/s12889-023-15110-2 (PMC9889245; doi:10.1186/s12889-023-15110-2)
Supplement: Supplementary file 3 — Additional file 3: Checking goodness-of-fit. Table 4. Average Bayesian p-values. [file 12889_2023_15110_MOESM3_ESM.docx]

## Additional file 3: Checking goodness-of-fit

If the model is adequate, it is expected that the posterior predictive distribution is similar to observed data. The posterior predictive p-values close to 0 and 1 suggest the predicted data by the selected model fall into one the extreme tails of the distribution, thus indicating the lack of fit. Table 4 shows that only a small percent of LTLAs in each year (less than 11%) have extreme posterior predictive p-values, suggesting that the specified model is adequate for data. In addition, the average p-value for each year is close to 0.5, confirming that the model can adequately generate predicted data.

Table 4. Average Bayesian p-values

| **Year** | **Average p-value** | **LTLAs with extreme p-values (%)** |
| --- | --- | --- |
| 2011 | 0.54 | 4.4 |
| 2012 | 0.53 | 5.7 |
| 2013 | 0.55 | 7.0 |
| 2014 | 0.54 | 5.4 |
| 2015 | 0.54 | 9.5 |
| 2016 | 0.55 | 5.4 |
| 2017 | 0.55 | 7.6 |
| 2018 | 0.55 | 8.9 |
| 2019 | 0.55 | 7.9 |
| 2020 | 0.56 | 11.1 |

## 
