# Supplementary material for: A decade of child pedestrian safety in England: a bayesian spatio-temporal analysis
Source: BMC Public Health. 2023 Feb 1;23:215. doi: 10.1186/s12889-023-15110-2 (PMC9889245; doi:10.1186/s12889-023-15110-2)
Supplement: Supplementary file 4 — Additional file 4: LTLA-specific time trends. Figure 8. LTLA-specific time trends. Figure 9. Time trend of local authorities where the crash incidence exceeds the average national value over time. Figure 10. Time trend of local authorities where the crash incidence goes below the average national value over time. [file 12889_2023_15110_MOESM4_ESM.docx]

## Additional file 4: LTLA-specific time trends

The LTLA-specific time trends are given by exp($\xi_{t}+\delta_{it}$) and are displayed in comparison to the national time trend in Figure 8. The fluctuation of LTLA-specific trend is due to unusual temporal trends in some local authorities partly due to unmeasured/unobserved factors (e.g., local risk factors or locally targeted interventions). In other words, after adjusting for the effect of covariates and main spatial and temporal effects, there are still some variabilities than can be explained through the interaction term.


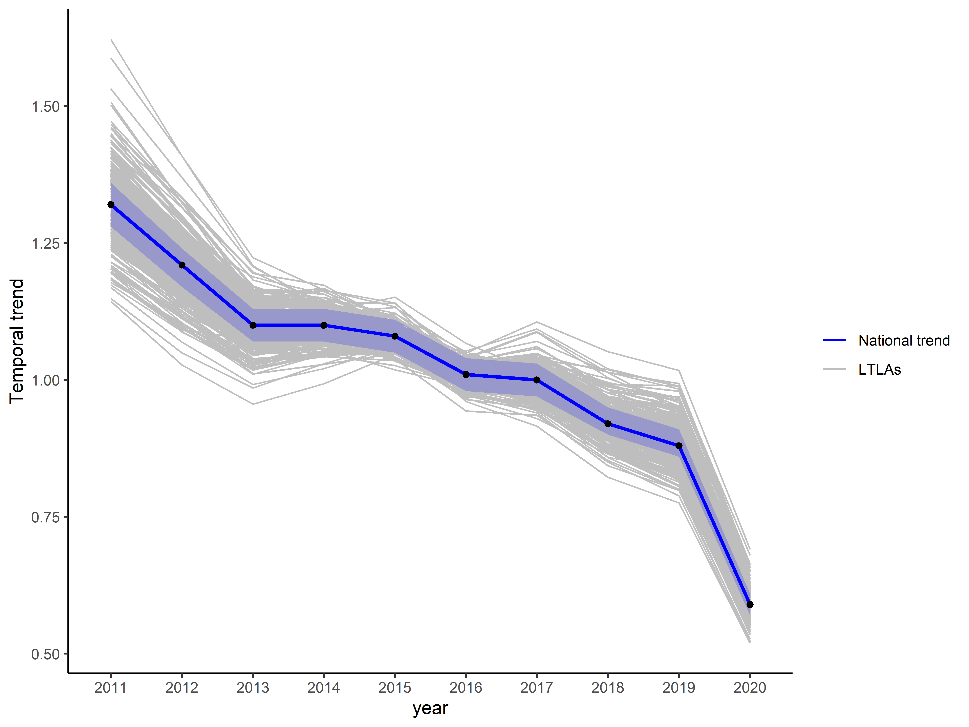


Figure 8. LTLA-specific time trends

To have a better understanding of time trends for local authorities, we calculated the probabilities that child pedestrian crashes in higher than the national one in each LTLA for each year. Figure 9 displays local authorities where the crash incidence was not likely (<20% probability) to exceed the national one in 2011 but the trend reverses over time such that these local authorities become highly probable (>80%) to have a larger number of crashes than the average national number of crashes. Figure 10 displays a contrary situation, where road safety conditions were improved, and the crash incidence became less than the national crash incidence over time.


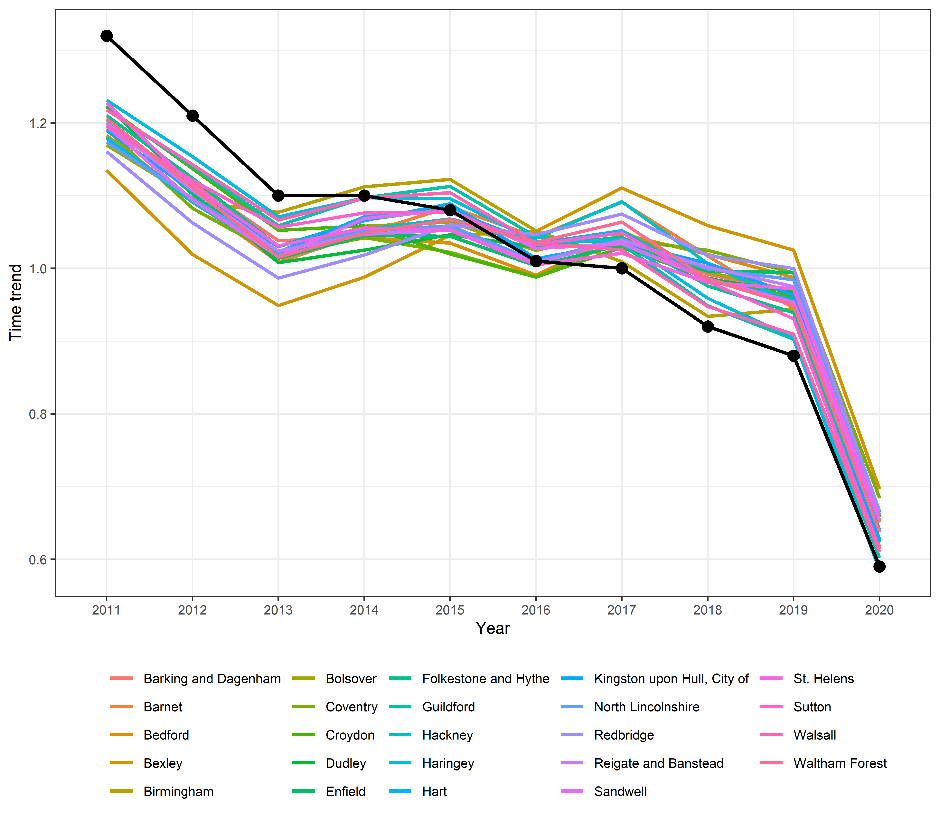


Figure 9. Time trend of local authorities where the crash incidence exceeds the average national value over time


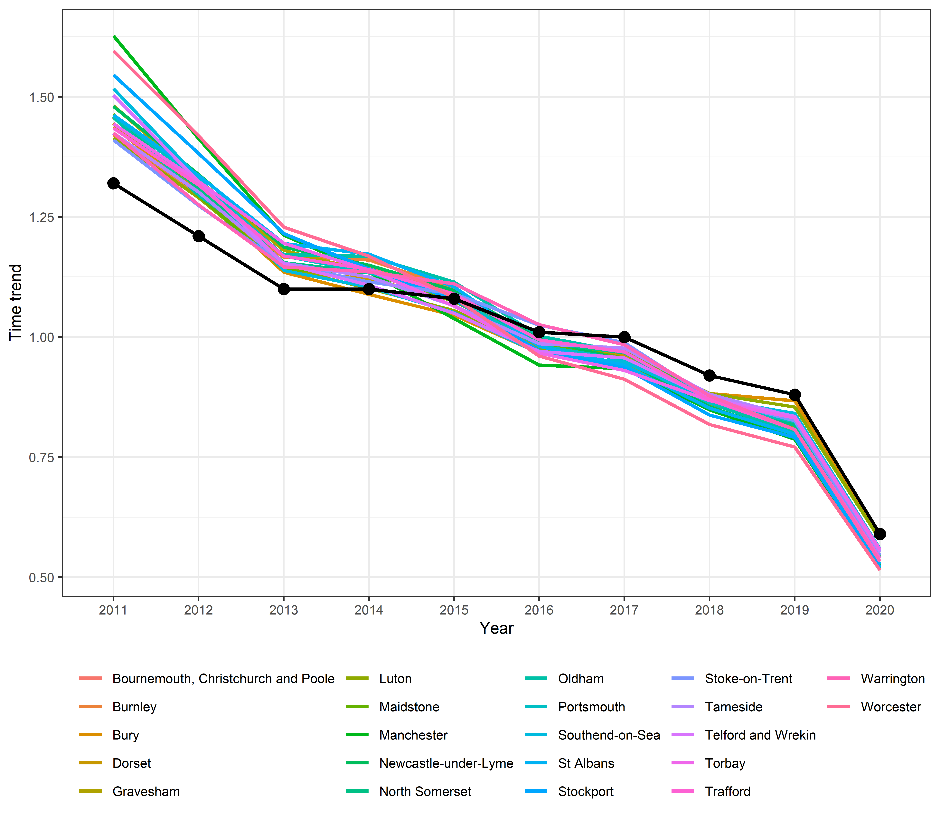


Figure 10. Time trend of local authorities where the crash incidence goes below the average national value over time
